# Convolutional neural networks for brain tumour segmentation

**DOI:** 10.1186/s13244-020-00869-4

**Published:** 2020-06-08

**Authors:** Abhishta Bhandari, Jarrad Koppen, Marc Agzarian

**Affiliations:** 1 Townsville University Hospital, Townsville, Queensland Australia; 2 Department of Anatomy, James Cook University, Townsville, Queensland Australia; 3 South Australia Medical Imaging, Flinders Medical Centre, Adelaide, Australia; 4 College of Medicine & Public Health, Flinders University, Adelaide, Australia

**Keywords:** Glioblastoma, Convolutional neural network, Artificial intelligence, Segmentation

## Abstract

The introduction of quantitative image analysis has given rise to fields such as radiomics which have been used to predict clinical sequelae. One growing area of interest for analysis is brain tumours, in particular glioblastoma multiforme (GBM). Tumour segmentation is an important step in the pipeline in the analysis of this pathology. Manual segmentation is often inconsistent as it varies between observers. Automated segmentation has been proposed to combat this issue. Methodologies such as convolutional neural networks (CNNs) which are machine learning pipelines modelled on the biological process of neurons (called nodes) and synapses (connections) have been of interest in the literature. We investigate the role of CNNs to segment brain tumours by firstly taking an educational look at CNNs and perform a literature search to determine an example pipeline for segmentation. We then investigate the future use of CNNs by exploring a novel field—radiomics. This examines quantitative features of brain tumours such as shape, texture, and signal intensity to predict clinical outcomes such as survival and response to therapy.

## Keypoints

- Convolutional neural networks simply involve analysing features derived from the image to perform tasks such as segmenting tumours.
- This initially involves training the network with a manually segmented dataset which then is poised to segment patient images.
- This has a role in segmentation of brain tumours such as glioblastoma and lower-grade astrocytomas.
- Segmented images can be further processed to predict clinical sequelae such as survival and response to therapy.

## Introduction

**Fig. 1:**
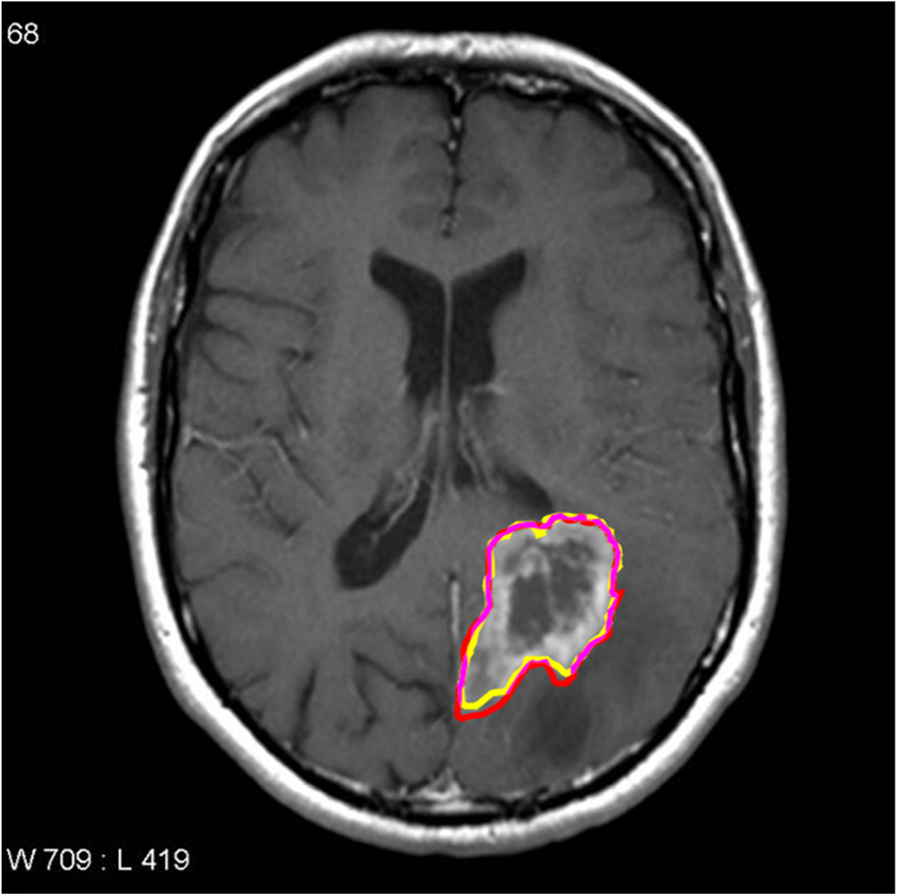
Manual anatomical segmentation by first (red) and second author (yellow) with intersection (purple). Image courtesy of A.Prof Frank Gaillard, Radiopaedia.org, rID: 22205

With the introduction of methods to quantitatively analyse gliomas with computational methods comes a new frontier for radiology. It is important for radiologists to be abreast of advances in machine learning. This has been recognised by the recent changes in the Royal Australian and New Zealand College of Radiologists (RANZCR) curriculum that incorporates machine learning into the part I applied imaging technology examinations [1]. Methods that incorporate quantitative analyses will add to the traditional visual analysis of images. An important step in the image analysis pipeline is the anatomical segmentation of regions of interest (ROI), for example, defining a volume of abnormal tissue from a background of normal tissue. This will allow for statistical analysis of features that is not visible by human perception [2]. For example, the field of radiomics is fast developing as a method of predicting survival times from imaging features such as shape of a volume of interest and texture and intensity of the voxel habitat. With the development of these methods comes a greater need for automated segmentation. Figure 1 shows inconsistencies in blinded manual segmentation of brain tumours by the first and second authors. A measure of consistency of image segmentation can be performed by the Sørensen–Dice coefficient, and this was calculated with the StudierFenster calculator (available at: http://studierfenster.tugraz.at/). This ranges from 0 to 1 with 1 having 100% consistency [3]. The value obtained from the segmentation by the first and second author was 0.91 which demonstrates the discrepancy in manual segmentation.

**Table 1:** CNN studies and main findings obtained from literature search

Author, year	Title	Main findings
Pereira, 2016 [30]	Brain Tumour Segmentation Using Convolutional Neural Networks in MRI Images	Small 3 × 3 kernels for convolution to combat overfitting. DSC—complete tumour, 0.78; core tumour, 0.65; and enhancing regions, 0.75
Arunachalam, 2017 [23]	An efficient and automatic glioblastoma brain tumor detection using shift-invariant shearlet transform and neural networks	Unique segmentation process involving SIST and NSCT transformation to convert the image into a multi-resolution image. Standard feature extraction occurs. Accuracy is reported at 99.8% for the proposed method
Havaei, 2017 [28]	Brain tumour segmentation with Deep Neural Networks	The TwoPathCNN (focusing on local and global paths) resulted in a DSC of complete segmentation, 0.85; core, 0.78; and enhancing, 0.73
AlBadawy, 2018 [31]	Deep learning for segmentation of brain tumours: Impact of cross-institutional training and testing	Training data on different institutions may produce dramatically different results. Therefore, CNNs need to be trained on data from the same institution
Hasan, 2018 [24]	A Modified U-Net Convolutional Network Featuring a Nearest-neighbour Re-sampling-based Elastic-Transformation for Brain Tissue Characterization and Segmentation	Traditional U-net ‘deconvolves’ the voxels rather than convolving. For this study, the deconvolution layer is substituted with an upsampling layer which passes through two convolution layers, an upsampling layer followed by augmentation by elastic transformation. DSC increased from 0.86 to 0.87.
Naceur, 2018 [29]	Fully Automatic Brain Tumour Segmentation using End-To-End Incremental Deep Neural Networks in MRI images	Incremental technique based on DSC which ‘learns’ features of scan until no features are learnt that increase DSC. This is iteratively refined. The DSC for this model is whole tumour, 0.89; tumour core, 0.76; and enhanced tumour, 0.81
Perkuhn, 2018 [22]	Clinical Evaluation of a Multiparametric Deep Learning Model for Glioblastoma Segmentation Using Heterogeneous Magnetic Resonance Imaging Data from Clinical Routine	Evaluation of DeepMedic architecture. DSC—whole tumour, 0.86; contrast enhanced tumour, 0.78; and necrosis, 0.62
Chang, 2019 [27]	A mix-pooling CNN architecture with FCRF for brain tumour segmentation	For global context, a fully connected conditional random field was combined to the CNN. DSC of complete tumour, 0.80; core tumour, 0.75; and enhancing, 0.71
Sundararajan, 2019 [25]	Convolutional Neural Network Based Medical Image Classifier	Segmentation by the watershed algorithm as opposed to manual segmentation for training sets improved accuracy from 82 to 89%. DSC is not reported
Chang 2019 [19]	Automatic assessment of glioma burden: a deep learning algorithm for fully automated volumetric and bi-dimensional measurement	Skull-stripping was superior to other methods proposed in the literature. FLAIR hyperintensities relating to oedema were able to be delineated in a multi-institutional context, pre- and post-operatively. DSC was 0.917 for the FLAIR volume

As an example of machine learning, this educational paper will examine the use of convolutional neural networks for low-grade diffuse astrocytoma (World Health Organization grade 2) and high-grade (World Health Organization grade 4) glioblastoma—also known as glioblastoma multiforme (GBM) segmentation. Convolutional neural networks (CNNs) are a unique machine learning structure originally modelled on the human visual cortex [4]. The brain was studied due to the abundance of segmentation methods that are already available and well established in the literature [5]. Machine learning is fast developing and is exponentially being represented at international conferences [6]. An educational perspective is needed for radiologists. This paper provides a novel balance between education and a state-of-the-art review on convolutional neural networks in glioblastoma.

To better understand CNNs, artificial neural networks will be reviewed briefly as this is a simple introduction for understanding neural networks such as CNNs. Artificial neural networks involve inputs which feed into a hidden layer which has biases or weightings associated with it and outputs which change as the machine ‘learns’ from a dataset to produce the expected result [7]. Further details will be provided in this paper.

**Fig. 2:**
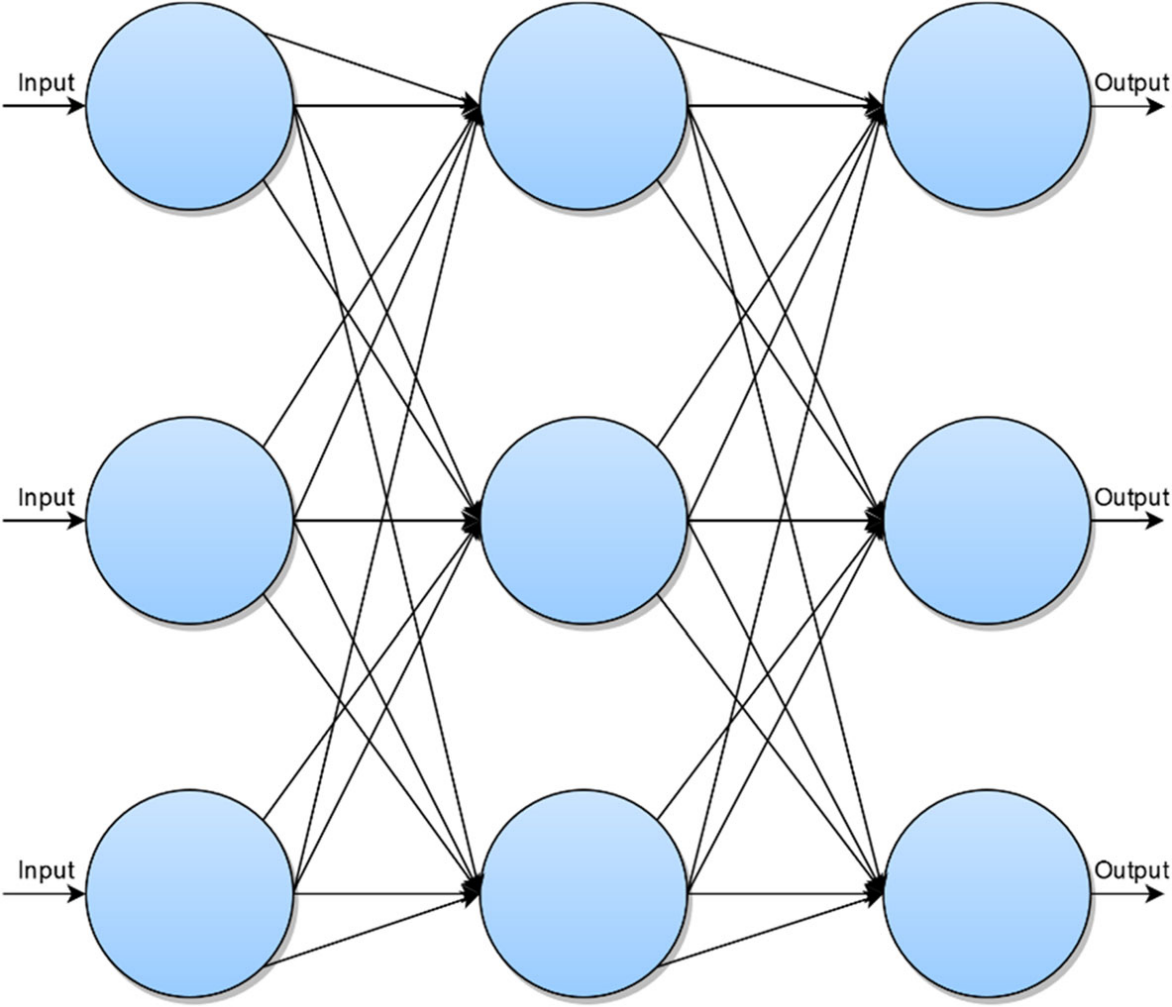
Diagrammatic representation of a convolutional neural network

From Fig. 2, each blue circle represents a node or neuron from which the name ‘neural network’ is derived from. There is an input to each neuron. The arrows or ‘axons’ represent the connection between neurons. The result is an output which generates an approximation of the image which is iteratively refined [8].

The nodes receive an initial input from a data source—as seen in Fig. 2. This is then fed into the next neuronal layer and given an initial weighting. This middle ‘*hidden*’ layer can be repeated a multitude of times. This is then fed into the output node, and this produced the desired result. However, this needs to be refined further, and one loop or *iteration* is not sufficient for the generation of an optimal output. This is where the novelty of a neural network comes in. In order to refine the output of the nodes, the weightings are changed. Thus, through iteration, the nodes are given different weightings. Based on these weightings, the output can changed through each iteration and eventually an output that reaches the desired result can be produced. Thus, neural networks provide a means of optimising an initial data input via weighting certain aspects of the input to produce an optimal result.

There are various other segmentation methods detailed elsewhere [9]. Some of the notable segmentation models are:

- Thresholding method—as the name implies, voxels above a threshold are classified as belonging to the tumour [10].
- Edge-based method—changes in the intensity between edges of voxels are used as the boundaries of the tumours [11].
- Region growing method—a seed voxel is inputted into the segmentation; from this seed, voxels that are similar are classified as belonging to the tumour [12].
- Watershed algorithm—this is a unique segmentation method whereby the voxel intensities or gradients are represented by a topographical map similar to those seen in geography. Based on the ‘steepness’ of the map, a boundary is assigned [13].
- Atlas method—a tumour free reference MRI is used to segment the MRI containing the tumour volume [14].

The advantages of the convolutional neural network are the fact that it provides optimal accuracy of segmentation. However, this is at the cost of computational load [9]. With advances in computation, the implementation of convolutional neural networks and refinement of the structural segmentation of brain tumours can be enhanced.

Diffuse astrocytic and oligodendroglial tumours are the commonest types of primary brain tumours in adults. Such tumours are classified on the basis of histological features such as cytological atypia, anaplasia, mitotic activity, microvascular proliferation, and necrosis by the World Health Organization as either grade 2, grade 3, or grade 4 with grade 4 having the worst prognosis. Glioblastoma (also known as glioblastoma multiforme or GBM) is the most frequently encountered grade 4 diffuse astrocytic and oligodendroglial tumour in adults [15]. Glioblastoma is a major public health concern. Zinn et al. [16] demonstrated that out of a cohort of 21,783 patients, the survival time without therapy has been shown to be 1 month; however, even with gross total resection, survival has been found to be only 11 months in the same population group. The cause of death is often not related to direct GBM complications. Diseases of the heart, pneumonia, influenza, stroke, and infections remain the top causes of death [17]. The accurate segmentation of GBM is a key step to allow for computational analysis.

The aims of this educational paper are to (1) describe machine learning as it pertains to neural networks, (2) describe current trends in low-grade diffuse astrocytoma and GBM segmentation with convolutional neural networks, and (3) describe potential uses and future directions.

## Machine learning and convolutional neural networks

**Fig. 3:**
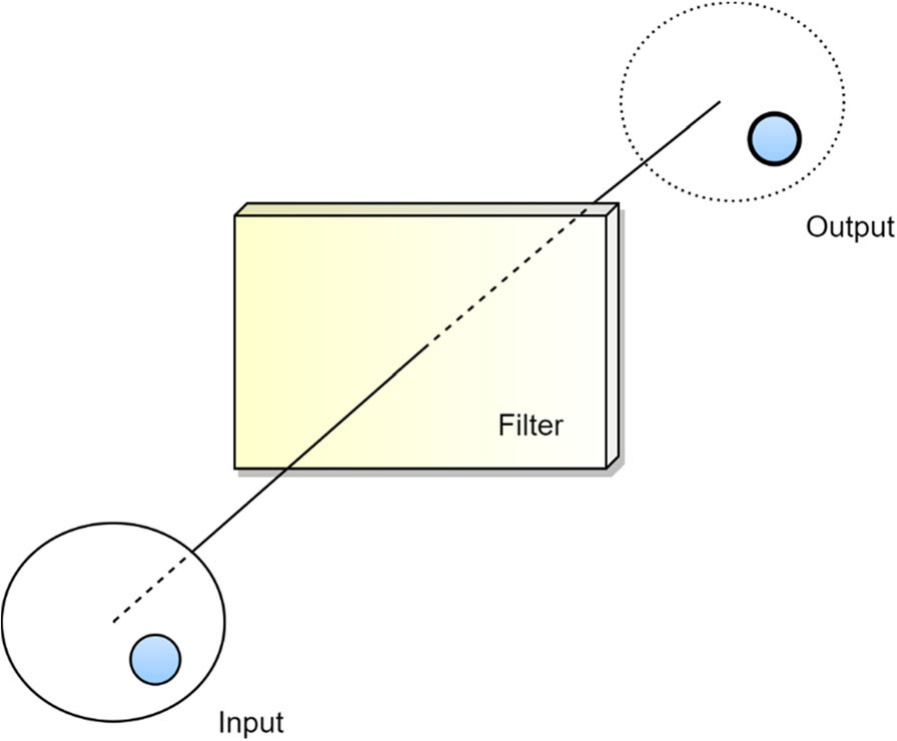
Filtration of input through convolution

CNNs work by using an input, convoluting this input with a filter (also termed a kernel) and giving an output. Figure 3 demonstrates the general overview of this process in a graphic, although additional steps are needed to produce a fully functional CNN. The network is first trained to a dataset. This is termed machine learning. This data training works by cycling or *iterating* through the training dataset multiple times and weighting the outputs or *applying a bias* on the artificial neurons based on the how close the output is to the expected output. With each iteration, the biases change to eventually obtain an output that is close to the expected output [8].

### Improving the output

To improve the output and develop the CNN, additional steps are needed. This involves overfitting correction, data augmentation, pooling, and the application of rectified linear unit (ReLU). Further details are below:

*Overfitting* refers to the fact that the neural network may be overtrained to the training dataset and produce inferior segmentations. To account for this, the CNN needs to be trained somehow to recognise imprecise features of the input. Four example methods are described. These are data augmentation, dropout, batch normalisation, and pooling.

*Data augmentation* can be used to generate imprecise inputs. Similar to the software used to edit photographic images, MRI images can be cropped, zoomed, and rotated [18]. This reduces overfitting as the neural network will not recognise specific patterns within the input dataset based on morphological arrangements which are deemed irrelevant between images. *Dropout* is a method whereby nodes are temporarily ‘dropped’ in the convolutional neural network in order to produce imprecision within the dataset. *Batch normalisation* is a method employed to reduce the weighting power of nodes that have a high bias. This allows for generalisability on other datasets as these high weights may be associated with specific precise features within the training set.

*Pooling* is where the input image is downsampled or the resolution is degraded in order to train the CNN to identify features that are imprecise. In other words, it reduces the chance of the network identifying insignificant details. For example, oedema on T2-FLAIR varies between patients. If the CNN was not pooled, it may pick up non-specific details from the training dataset resulting in imprecise segmentation of oedema from the tumour during validation [8].

From the filtered data, the image is approximated using a linear function. Given the heterogeneity of biological patterns, this only provides a rough approximation of the actual image. There needs to be a correction to account for non-linearity within the image. A rectifier can be applied which approximates for this non-linearity within the dataset. An example unit that applies the rectifier is called the ReLU [8].

**Fig. 4:**
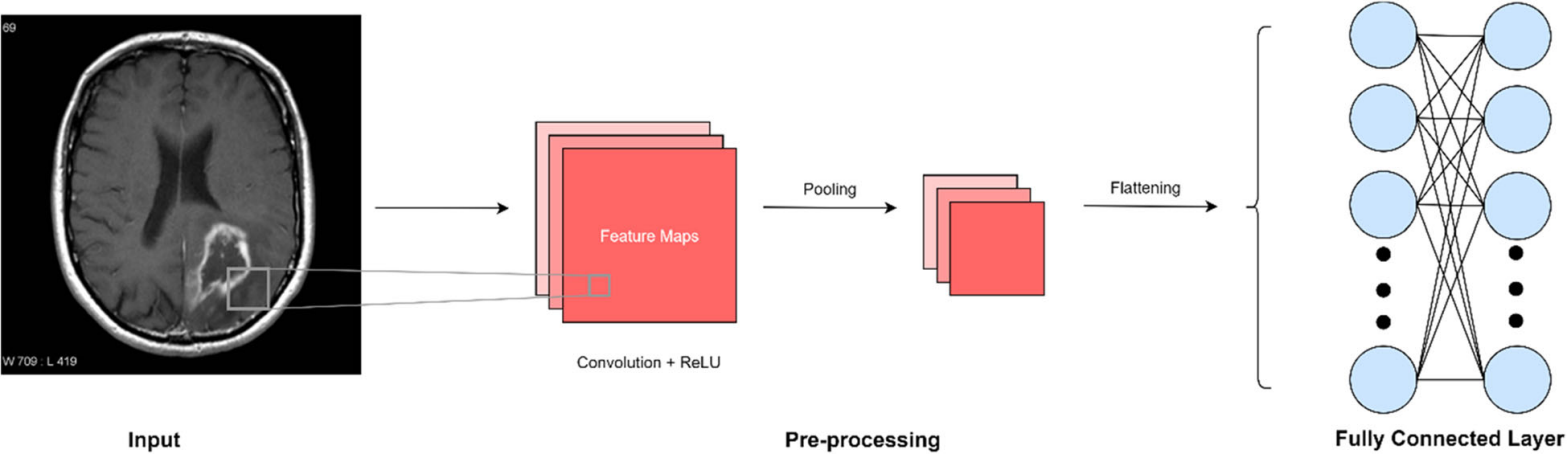
Input into a single node within a convolutional neural network

The network is then flattened from the feature map into a column for input into the neural network. In simple terms, there is the (1) input, (2) convolution with non-linearity correction through ReLU, (3) overfitting correction (in Fig. 4, we demonstrate pooling), (4) flattening, and (5) insertion into the neural network. The steps for this process are detailed in Fig. 4.

**Fig. 5:**
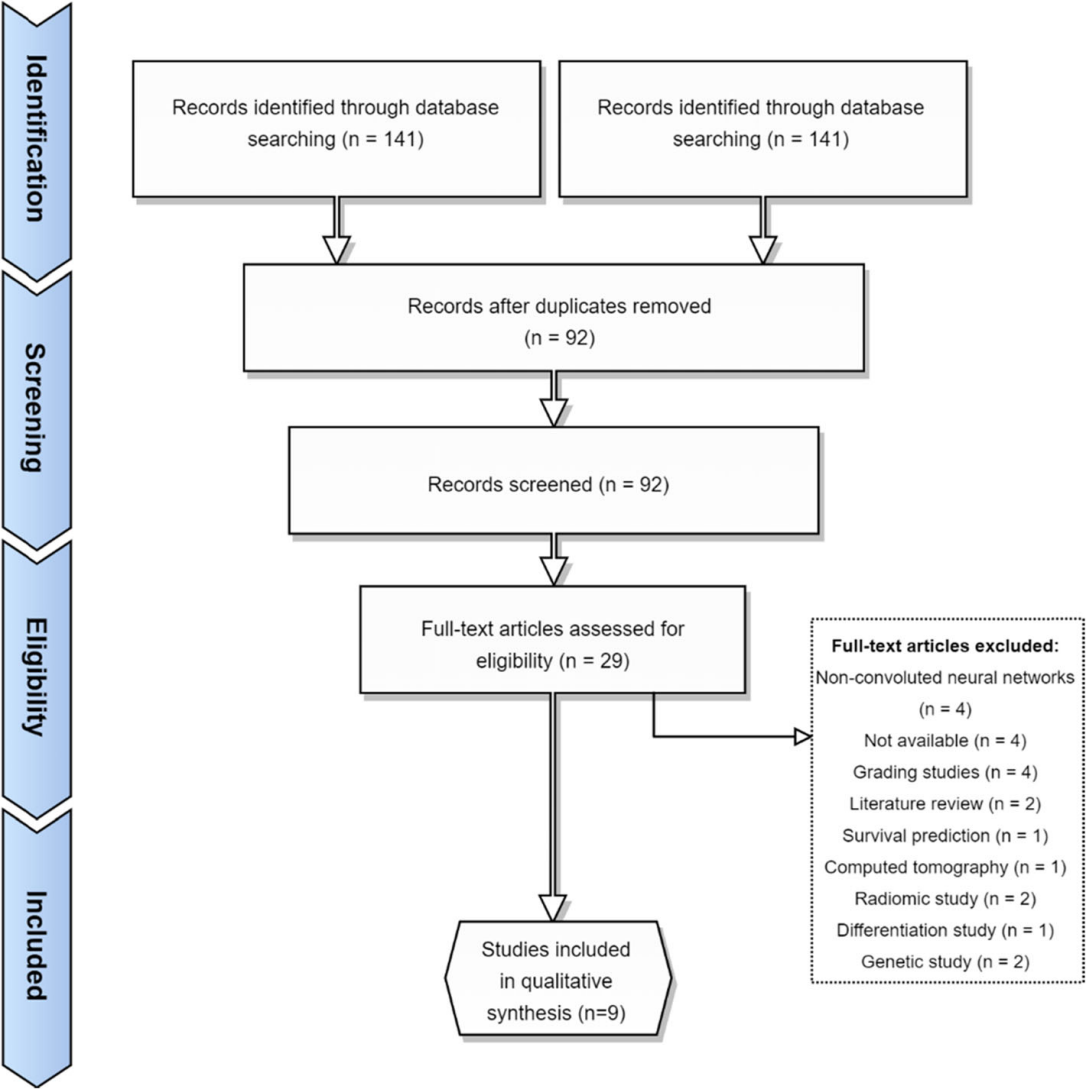
PRISMA flowchart for search performed on the 24 October

## Literature review process

As per the Preferred Reporting Items for Systematic Reviews and Meta-Analyses (PRISMA), a literature review was performed on Web of Science, Scopus, and PubMed using the search terms: (neural AND network*) AND (GBM OR “glioblastoma” OR astrocytoma) AND segment*. We found 18 articles from PubMed, 72 from Scopus, and 51 from Web of Science. After duplicates were removed, 92 articles remained. For a broad scope, we included all studies that examined convolutional neural networks in MRI brains the past 5 years. We excluded conference abstracts, non-English papers, reviews, and genomic papers. Microscopy papers were excluded. Grading studies and differentiation studies were also excluded on the basis that these studies did not address segmentation directly. After the inclusion and exclusion criteria were applied, 29 studies remained. Full texts were reviewed. Four studies were excluded on the basis that they were non-convoluted neural networks, 3 studies were excluded since they were not available, and 3 grading studies, 2 reviews, 2 purely genetic studies, 1 survival prediction study, 1 computed tomography study, 2 radiomic studies, and 1 which looked at the differentiation between different tumours were also excluded. This is summarised in Fig. 5.

**Fig. 6:**
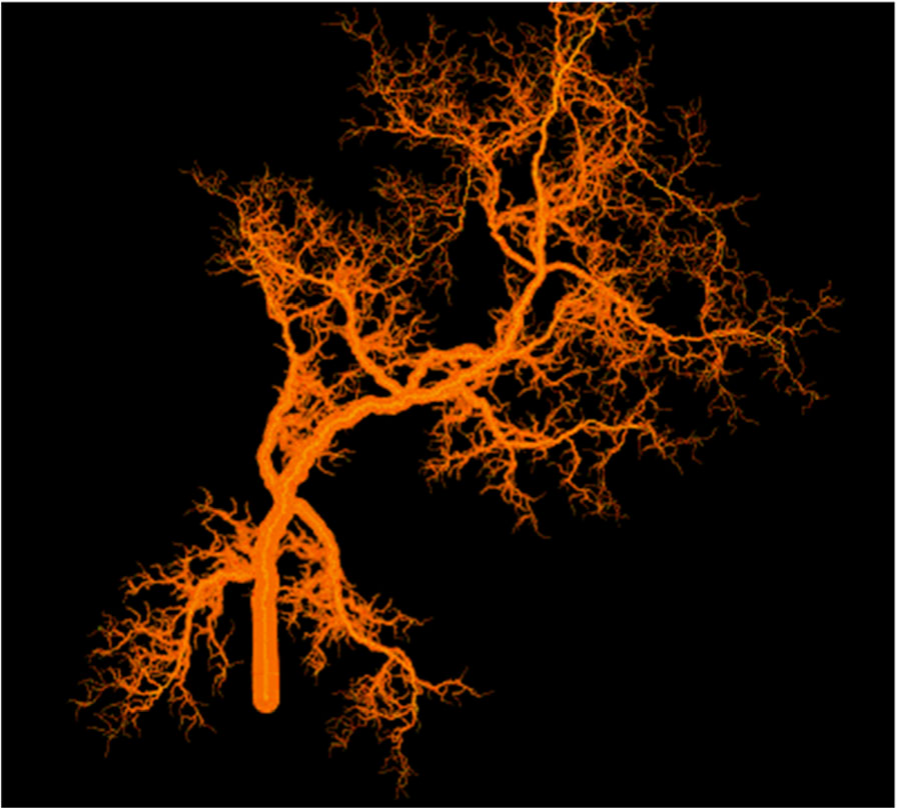
Generation of fractal model of tumour microvasculature through FracLac [37]

In addition to the 9 studies found by the data search, a hand search revealed one additional study [19]. Therefore, *n* = 10 studies were used in the qualitative analysis.

### Literature findings

Convolutional neural networks represent a growing field within the literature. Our search found 10 studies that detailed the methodology of segmentation involving convolutional neural networks. The methodology examined in this review will be divided into subsections relating each step of the segmentation process. The main findings will be reported. An example segmentation algorithm will be proposed based on the findings from the literature. The main output measure is the Sørensen–Dice coefficient (DSC) which is calculated as follows [3]:

DSC = $$ \frac{2\mid X\cap Y\mid }{\left|X\right|+\mid Y\mid } $$

The *X* represents the cardinal elements in the first set (automatic segmentation set), and *Y* represents the second set—generally the manually segmented set that the automatic segmentation set is tested against. The ⋂ symbol represents where the segmentations intersect. Where the DSC is not reported, the accuracy will be used. Table 1 reports the main findings of studies involving CNNs.

For the training set, most studies used the Multimodal *Br*ain *T*umour *S*egmentation (BraTS) benchmark which is a dataset of manually segmented MRIs containing high-grade and low-grade gliomas set up by the Medical Image Computing and Computer-Assisted Interventions (MICCAI) [20]. Only one study [21] used a training set from their own institution. This study negated the need for initial manual segmentation due to using a watershed algorithm which automatically segmented the training dataset. This improved the accuracy of segmentation from 82 to 89% and could be used for the development of future CNNs.

Specifics of convolution layers (i.e. filtration of images) were not detailed extensively. This is partly because feature extraction involves multiple algorithms and multiple methodologies. For example, Perkuhn et al. [22] used 5^3^ kernels for feature extraction in four convolutional layers. It would be difficult to summarise such extensive and numerous convolution methodology.

Overfitting was done in a variety of ways. Three articles did not correct or did not report details of overfitting [23–25]. The majority of overfitting was done via down sampling [24, 26–29]. This involves reducing the resolution of the image in order reduce interpretation of irrelevant fine details within the training dataset. Pereira et al. [30] used a unique method of overfitting correction whereby they used augmentation by 90° rotation on the training dataset.

For non-linearity correction, different algorithms were used. This includes leaky rectifier units [30], max-out non-linearity [28], noisy rectifier linear units [26], rectified linear units [29], and parametric rectified linear unit which is a modification of the traditional rectified linear unit. One study used 3 corrections—leaky rectifier units, Softmax function, and hyperbolic tangent function [27]. For two studies, the non-linear correction applied was not reported [23, 25].

From the literature search, an example segmentation process is devised. The segmentation process should be fully automated and in an ideal situation be performed in institutions with the same scanner/imaging protocols given discrepancies would affect the segmentation process [26]. However, this would negate the generalisability to other contexts and CNNs need to be optimised for the multi-institutional context. Normalisation of images could be done by methods proposed in the BraTS [20] segmentation challenge. This was done by standardising multi-institutional images to 1 × 1 × 1 mm voxel parameters which were then skull stripped. This initial training input can be done via the watershed algorithm which has been shown to have superior segmentation potential than manual segmentation [25]. Nearest-neighbour Re-sampling-based Elastic-Transformation (NNET) U-Net deep convolution algorithm suggested by Hasan et al. [24] can be applied for the initial filter as this has shown to increase the DSC. Overfitting can be improved by using the ELOBA_*λ* algorithm proposed by Naceur et al. [29] which has shown a DSC of 0.89, 0.76, and 0.81 for the whole tumour, tumour core, and enhanced tumour respectively. FLAIR volume can be segmented with the methods proposed by [19] as DSC has been reported as 0.917 for volume of FLAIR hyperintensity. Non-linearity correction has not been extensively studied, so no recommendations can be made. The main limitation is the computing power given the intricate processes involved in each step.

## Clinical applications and future directions

The main application of brain tumour segmentation in the clinical sphere is quantitative image analysis. Brain tumours have traditionally been analysed qualitatively through inspection, and this have given rise to an elaborate feature lexicon summarised by projects such as Visually AcceSAble Rembrandt Images (VASARI) [32]. Radiomics is a field focused on quantitative feature analysis. This field is focused on extracting voxel and volume features of the tumour habitat and predicting clinical outcomes such as survival. It has been used to grade tumours, evaluate response to therapy and predict genetic status of GBM—for example, isocitrate dehydrogenase (IDH) status in GBM, which is of clinical significance since negative status (or ‘wildtype’) implies a more aggressive tumour [33]. Segmentation and the processes involved in extraction can be translated into meaningful analysis in radiomics. Radiomic features such as shape, texture, intensity, and patterns of the microvasculature have been extensively studied in GBM [34].

Shape and texture represent the most extensively studied radiomic features. Filtration matrices that are applied during the convolution of images during the segmentation processes can subsequently be used to predict clinical outcomes such as survival. For example, in a study by Sanghani et al. [35], 2200 shape, volumetric, and texture features were extracted from 163 patients. Using kernels for each feature, i.e. shape, texture, and volume, prediction of overall survival was sorted into groups with short (< 10 months), medium (10–15 months), and long (> 15 months) term survival with 88.95% accuracy. In the same study, prediction of short (< 400 days) and long (> 400 days) was shown to have a higher accuracy of 98.7%. This demonstrates the novelty of radiomics in predicting clinical outcomes and the importance of translating segmentation algorithms into radiology.

Other applications are in the sphere of treatment planning, particularly for radiotherapy and targeted chemotherapy. Assessment of the tumour microvasculature can be performed by fractal analysis which assesses repeating patterns of the vasculature not easily represented by simple Euclidian geometry. Studies of tumour vasculature through susceptibility-weighted imaging (SWI) have shown a decrease in a measure of fractal capacity dimension post-treatment with an anti-angiogenic (bevacizumab) [36]. Figure 6 demonstrates the modelling of tumour vasculature in the programme FracLac [37]. This demonstrates the applicability of quantitative analysis to predict tumour response to therapy. Similarly, tumour volumes and features such as oedema, spiculation, and necrosis can be extracted to represent response to radiotherapy [38]. This demonstrates the novel use of segmentation processes to to aid in the production of meaningful results that have an impact on clinical practice.

Machine learning has an important role in the automation of quantitative image analysis. The dataset is trained using feature extractions on a training dataset which necessitates the need for feeding fixed parameters. Machine learning through multiple iterations can provide more accurate results than standard voxel-based manipulation methods. Survival prediction for classification into short, medium and long term survival has shown to have a moderate accuracy of up to 57.8% using ensemble learning (combination of multiple machine learning algorithms) in a study of 9 types machine learning models [39]. Further work is needed in this area to investigate the highest predictive machine learning models of survival, and this can be achieved by translation of segmentation features extraction and modelling into radiomics.

## Conclusion

Convolutional neural networks remain a growing area of research in automated tumour segmentation. It is important for radiologists to have a working knowledge of convolutional neural networks so that they are well positioned to deploy these tools in future clinical practice. A basic overview is provided in this paper that allows the reader to be well-informed in the world of automated segmentation. Clinical applicability to GBM is highly relevant as brain tumour segmentation from normal parenchyma is the first step in the utilisation of quantitative image feature analysis such as radiomics for prognostication, and treatment planning. Through the further development of segmentation techniques in brain tumours, this could be applied to other areas of radiology. Convolutional neural networks represent a growing field that will likely help radiologists provide more accurate care for their patients.

## Data Availability

Not applicable

## Abbreviations

BraTS: *Bra*in *T*umour *S*egmentation
CNN: Convoluted neural network
DSC: Sørensen–Dice coefficient
GBM: Glioblastoma multiforme
IDH: Isocitrate dehydrogenase
MICCAI: Medical Image Computing and Computer-Assisted Interventions
MRI: Magnetic resonance imaging
NNET: Nearest-neighbour Re-sampling-based Elastic-Transformation
RANZCR: Royal Australian and New Zealand College of Radiologists
ReLU: Rectified linear unit
ROI: Region of interest
SWI: Susceptibility-weighted imaging
WHO: World Health Organization
